# Chromatin states and nuclear organization in development — a view from the nuclear lamina

**DOI:** 10.1186/s13059-015-0747-5

**Published:** 2015-08-25

**Authors:** Anna Mattout, Daphne S. Cabianca, Susan M. Gasser

**Affiliations:** Friedrich Miescher Institute for Biomedical Research, Maulbeerstrasse 66, CH-4058 Basel, Switzerland; University of Basel, Faculty of Natural Sciences, Klingelbergstrasse 50, CH-4056 Basel, Switzerland

## Abstract

The spatial distribution of chromatin domains in interphase nuclei changes dramatically during development in multicellular organisms. A crucial question is whether nuclear organization is a cause or a result of differentiation. Genetic perturbation of lamina–heterochromatin interactions is helping to reveal the cross-talk between chromatin states and nuclear organization.

## Introduction

Since the earliest days of microscopy, there have been studies indicating that chromatin and chromosomes are not randomly distributed in interphase nuclei [1]. We now know that the distribution of chromosomes into distinct territories, the clustering of specifically modified chromatin with itself and the nuclear periphery, and the long-range contacts that form between control regions and promoters are all relevant features of nuclear organization [2, 3]. Other aspects of genome organization within the nucleus include the spatial sequestration of origins of replication into replication foci, and the clustering of promoters into sites of active transcription [4].

One of the most pronounced and conserved features of genome organization — particularly in the nuclei of differentiated cells — is the close proximity of heterochromatin to the lamina or nuclear envelope (NE) [2, 5]. Nonetheless, it has been surprisingly difficult to identify the proteins responsible for this perinuclear sequestration. Although nuclear lamins are candidates, their link to chromatin is almost certainly indirect and their chromatin-organizing function is often redundant with that of other proteins. Indeed, single-celled organisms lack lamins altogether yet nonetheless are able to tether silent chromatin to the nuclear envelope using specialized anchors. Some of these are species specific and others are highly conserved [6].

To demonstrate the physiological relevance of potential signals and anchors in nuclear organization, it is essential to use genetic approaches, namely to mutate the relevant gene and examine the consequences of this in vivo. From such studies in mice, it has been shown that lamin A/C and the lamin B receptor (LBR) contribute to heterochromatin localization at the NE in a partially redundant manner in differentiated tissues [7, 8]. Further support for a role of lamin A/C in tissue-specific gene regulation comes from the identification of 16 late-onset, tissue-specific diseases in man caused by over 400 different point mutations in the gene *LMNA*, which encodes both lamin A and lamin C [9, 10]. Whether these degnerative laminopathic phenotypes stem from altered subnuclear chromatin organization remains to be seen.

In this review, we focus specifically on genetic data that link the three-dimensional organization of the genome to gene expression and cell-type commitment during cell differentiation. Because chromatin modifications influence both genome function and nuclear organization, we first review the changes in chromatin that correlate with cell differentiation and then summarize new insights into factors that determine the distribution of chromatin in the nucleus. Finally, we examine a few examples of the diverse effects that stem from mutations in lamin A/C.

## Genomic marks: pluripotent versus differentiated epigenomic landscapes

In principle, each cell of a multicellular organism has the same genetic material. Yet cells manifest strikingly different cell morphologies and functions, reflecting their distinct patterns of gene expression. Accompanying the active induction of tissue-specific genes is an accumulation of heterochromatic domains that are stably repressed in terms of transcription. Whereas constitutive heterochromatin remains compact throughout the cell cycle and in every cell type, facultative heterochromatin contains tissue-specific genes that are selectively repressed, reflecting cell-type-specific restriction of gene expression. Chromatin distribution in the nucleus is also distinct for each differentiated cell type [8], yet, at present, we understand only a few basic rules. Generally, transcriptionally repressed heterochromatin clusters away from active genes, sequestered either by the nucleolus or the nuclear periphery, whereas active chromatin tends to be internal or at nuclear pores [2, 11]. The signals that ensure cell-type-specific distribution of chromatin domains are the focus of ongoing studies.

It is clear that chromatin distribution in the nucleus is not only affected by silent chromatin. The expression of developmentally regulated genes is determined by transcription factors that bind both to promoters near transcription start sites and to distal enhancers. These factors often mediate enhancer–promoter looping and recruit histone modifiers, which in turn alter the long-range folding of the chromatin fiber [12]. Such interactions determine which promoters are active in a given cell type, and the ensuing chromatin status helps define the subnuclear position of genes [11, 13]. Therefore, the study of nuclear organization must include an analysis of histone modifications and their distribution.

The study of genome-wide chromatin modifications has been promoted by a number of new methods (Box 1). Chromatin immunoprecipitation (ChIP) coupled with microarray or sequencing analysis (ChIP-chip, ChIP-seq, MeDIP), as well as bisulfite-seq for CpG methylation, reveals epigenetic marks genome-wide [14]. The mapping of long-range interactions between distant sequences is scored by ‘chromosome conformation capture’ technologies (3C, 4C or HiC; Box1) [15], and the DNA adenine methyltransferase-fusion identification (DamID) technique [16] allows one to specifically methylate adenine residues in sequences that contact a protein of interest — for example, a nuclear lamin (Box 1). These methods have been applied to in vitro differentiation systems, such as the differentiation of mouse embryonic stem cells (ESCs) into neuronal progenitor cells (NPCs) and differentiated neuronal cell types [17]. ESCs are also compared with independently obtained differentiated cell lines, or mouse embryonic fibroblasts (MEFs), even though ESCs themselves can have different levels of pluripotency. Rigorous conclusions about epigenetic marks and gene expression will require the application of these techniques to stem cells and tissues in living organisms.

Nonetheless, a number of conclusions can be drawn from the mouse ESC system. It has been shown that DNA methylation on CpG residues increases at a subset of tissue-specific promoters that become silenced during tissue differentiation. Interestingly, these de novo methyl-cytosine (meC) targets are often found on nucleosomes that were initially bivalently modified in the committed precursor stage — carrying both the active histone H3 lysine 4 trimethylation (H3K4^me3^) and the repressive histone H3 lysine 27 di- or trimethylation (H3K27^me2/me3^) mark [18, 19]. Prominent differentiation-associated changes in CpG methylation also occur at enhancers, which tend to lose methylation upon activation [14]. Importantly, it was shown that CpG methylation is targeted to sites by sequence-specific DNA-binding factors [20], just like the targeting of silent information regulatory (SIR)-mediated repression in budding yeast through silencers (reviewed in [21]).

Naturally, histone modifications correlate with ESC differentiation [22, 23]. In general, unmethylated CpG island promoters carry H3K4 methylation in all cell types when active, whereas those that are transcriptionally inactive in ESCs have both H3K4^me3^ and H3K27^me3^ [23]. In this case, it is unclear whether H3K27^me3^ itself is repressive, as the loss of the histone methyl transferase (HMT) complex depositing this mark [polycomb repressor complex 2 (PRC2)] had almost no effect on gene expression [24]. Nonetheless, H3K27^me3^ levels fluctuate a great deal at specific promoters during ESC differentiation — hundreds of promoters gain this mark, whereas as many others lose it, during the transition from ESC to NPCs, and from NPCs to differentiated neurons [18, 19]. When bivalent promoters lose H3K27^me3^, they generally become activated in later differentiated states, suggesting that polycomb keeps different sets of genes poised for appropriate expression at later stages of differentiation [22, 25–28].

A general hallmark of transcriptionally silent heterochromatin is methylation of histone H3 lysine 9 (H3K9). It is a matter of debate whether the overall amount of the heterochromatic histone H3K9 di- and tri-methylation increases during differentiation of ESCs [29, 30]. Lienert and colleagues observed no global increase in histone H3K9^me2^ during ESC-to-neuron differentiation, although localized changes were found at specific genes [31]. By contrast, Wen and colleagues reported that histone H3K9^me2^ coverage in large chromatin domains increased from a range of 17.5–24 % in pluripotent human stem cells to a range of 39.3–44.8 % in differentiated cell lines [29]. The bioinformatics normalization procedure used has been disputed [30], yet it is agreed that large domains of H3K9 methylation exist. Perhaps because the bulk of the H3K9^me2/me3^ is associated with repetitive DNA (satellites, dispersed long terminal repeats [LTRs], retroviral elements and simple repeats, which constitute from 60–70 % of a mammalian genome [32]), the quantity of H3K9 methylation deposited on tissue-specific genes seems relatively insignificant. Nonetheless, it could have a major impact on gene expression [25]. Finally, one should note that size-selection of fragments during ChIP-seq library preparation could lead to a bias against the inclusion of large H3K9^me^-containing heterochromatic domains.

The imaging of condensed heterochromatin and chromocenters by microscopy confirms that there are major changes in heterochromatin during differentiation: dense-staining foci of heterochromatin are less obvious in undifferentiated than in differentiated ESCs and are less frequently perinuclear [33–36]. Similarly, the inactivated X chromosome in mammalian female somatic cells [37], like major and minor satellite repeats, becomes more compact as cells differentiate [38–41]. Consistently, undifferentiated or pluripotent ESCs tend to have fewer and less compact foci of the major H3K9^me2/me3^ ligand, heterochromatin protein 1α (HP1α) [38, 42]. Two other HP1 isoforms, HP1β and HP1γ, do not localize with heterochromatic chromocenters in undifferentiated cells but instead assume a diffuse nuclear distribution [43]. Surprisingly, ESCs derived from mice lacking HP1β failed to maintain pluripotency, showing a tendency to differentiate spontaneously into ill-defined ectoderm [43]. At the same time, differentiated cells with reduced H3K9 methylation or lacking HP1β were more readily reprogrammed into induced pluripotent stem (iPS) cells [35, 42]. This argues that both H3K9^me3^ and HP1β act as barriers to the reprogramming of differentiated cells [44–47]. Nonetheless, HP1β appears to play additional roles upregulating genes in ESCs as observed previously in *Drosophila* embryos [48].

In summary, the modulation of chromatin states during differentiation provides a basis for changes in nuclear morphology, as well as for changes in gene expression. In general, pluripotent genomes are less rigidly organized than differentiated states, as demonstrated both by biochemical and fluorescence recovery after photobleaching (FRAP) methods [38, 49, 50] and by nuclear morphology. A further, important functional change in chromatin that occurs during mouse ESC differentiation to neurons is an increase in late-replicating domains [51]. The changes in replication timing are cell-type-specific and correlate broadly with changes in transcription, as well as with the emergence of compact chromatin close to the nuclear periphery [36, 52]. How replication timing impacts differentiation remains to be explained, yet the spatial segregation of differentially timed replication events is an important hint.

## Multiple classes of chromatin in differentiated cells and contact with the nuclear lamina

In order to classify the chromatin states that exist in differentiated cells, several laboratories have used principal component analysis and/or hidden Markov models (HMMs) to analyze histone modifications and non-histone protein binding patterns. Genome-wide mapping data from *Drosophila* tissue culture cells were used to define chromatin classes by principle component analysis [53, 54], and five distinct types of chromatin were identified. These included three classes of silent chromatin: simple-repeat-associated HP1-bound chromatin; H1-associated and lamin-associated chromatin on silent tissue-specific genes; and polycomb-enriched silent domains [53]. Transcriptionally active chromatin fell into two classes: one enriched for histone H3 lysine 36 (H3K36) methylation and its ligand, Mrg15, and a second class being very early replicating and enriched for large regulatory protein complexes such as histone acetyltransferases and remodelers. A similar, but distinct, HMM approach has been applied to histone modifications mapped in differentiated human CD4^+^ T cells [54]. In this case, five classes each of euchromatin and heterochromatin were defined, and upstream regulatory sequences could be distinguished from coding regions based on their histone modifications [54]. In both studies, one major class of silent chromatin was associated with nuclear lamins.

## Genome organization and the nuclear lamina

The nuclear lamina is a meshwork of proteins that lies adjacent and anchored to the nuclear membrane. Its main structural components are the type V intermediate filament proteins lamin type A and lamin type B (Table 1; reviewed by Gruenbaum et al. [55]). The nuclear lamina also comprises a large variety of proteins that span the inner nuclear membrane (INM) called nuclear envelope transmembrane proteins (NETs), which are associated directly or indirectly with lamins (e.g., LBR, emerin, LAP2β, LEM-2) [56]. Importantly, the composition of the nuclear lamina differs among cell types and stages of differentiation [7, 8, 57, 58], and the NETs themselves show significant cell type specificity [59].

**Table 1:** The basic characteristics of the lamins

Lamin type	Genes (in mammals^a^)	Major isoforms/(minor isoforms)	Processing of the C-terminus	Isoelectric point (pK) and solubility in mitosis	Expression
A-type lamin	*LMNA*	Lamin A	Lamin A and AΔ10 have a CaaX motif that is farnesylated and ultimately cleaved with an additional 15 residues from the C-terminus	pK neutral	Lamins A and C are expressed in most somatic differentiated cells Lamin C2 is expressed in germ cells
		Lamin C	Lamin C and C2 have no CaaX motif	Soluble during mitosis	
		(Lamin AΔ10 Lamin C2)			
B-type lamin	*LMNB1*	Lamin B1	B-type lamins have a CaaX motif that is farnesylated and carboxymethylated on the cysteine, whereas the (aaX) part of the motif is removed	pK acidic	Lamins B1 and B2 are expressed in most or all somatic cells, but lamin B1 is notably absent from muscle cells. Lamin B3 is found in germ cells
	*LMNB2*	Lamin B2	The farnesyl group is essential but not sufficient for the peripheral localization of B-type lamins	Membrane-bound in mitosis	
		(Lamin B3)			

^**a**^The worm *Caenorhabditis elegans* has a single lamin gene (*lmn-1*) encoding a protein that has features of both A-type and B-type lamins

**Fig. 1:**
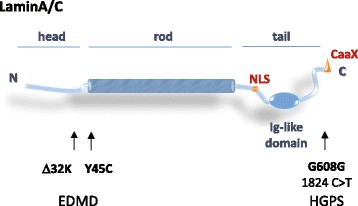
Lamin structure. A schematic sketch of a generic lamin protein, highlighting the important structural features. The N-terminal head domain is short and mostly unstructured, and also contains a conserved phosphorylation site flanking the rod domain, which is important for lamin polymer disassembly and reassembly during mitosis. Another phosphorylation site is situated at the other extremity of the rod domain. The central rod domain is mainly composed of α-helices, consisting of four coiled coils, interrupted by flexible linker domains. The rod domain is essential for the dimerization of lamin, which is the first step required for the assembly of lamin filaments. The C-terminal tail domain of lamin protein includes a structured immunoglobulin-like domain, structurally well conserved among species, as well as the evolutionarily conserved nuclear localization signal (NLS) and CaaX motifs ("C" stands for cysteine, "a" any aliphatic amino acid, and the identity of "X" determines the enzyme that acts on the protein). In lamin the motif is recognized by a farnesyltransferase. Arrows under the sketch indicate the position of the two EDMD causing mutations on the lamin protein discussed in the review, and of the most common HGPS (progeria) mutation G608G. Δ32K in mice corresponds to the deletion of the lysine 32, which corresponds to Δ46K in *C. elegans*. Y59C is a missense mutation at the beginning of the rod domain in *C. elegans* lamin, analogous to the 45C mutation in human lamin A/C. The hundreds of other mutations leading to laminopathies are spread almost all over the lamin protein [151]

Although lamin proteins (Fig. 1) are not essential for viability in non-dividing cells, nor in organisms with closed mitoses, the analysis of organisms lacking lamin A or lamin B clearly implicates lamins in nuclear organization and in cell integrity. In dividing cells of the worm *Caenorhabditis elegans* and in cultured human cells, B-type lamins are essential for successful cell division [60, 61]. Similarly, mouse embryos lacking B-type lamin have delayed mitoses and cumulative developmental defects [62–65]. Although B-type lamins can compensate for A-type lamins in mammalian cell division, *lmna* deficiency leads to perinatal death in humans, as well as in mice, which die shortly after birth owing to muscle and heart failure [66]. Importantly, the expression levels of lamin A/C increase upon cell differentiation, and, in tissues such as striated muscle, *lmna* point mutations can perturb nuclear shape, gene expression and mechanotransduction signaling, as will be discussed below [67].

The technique lamin-DamID (Box 1) has been used to map sequences genome-wide that interact with the nuclear envelope in multiple cell types. Initially, the group of Bas van Steensel identified approximately 500 genes in these lamin-associated domains (LADs) in the *Drosophila melanogaster* Kc cell line [68]. They went on to show that approximately 40 % of the genome of cultured human lung fibroblasts touches the lamina at least transiently, defining LADs that range in size from 0.1–10 Mb [69]. The average gene density within LADs is about half that of non-LAD regions, and most of the genes were silent or poorly expressed, as the regions are naturally AT-rich and gene-poor [9, 70]. Using either lamin DamID or LEM2 ChIP in *C. elegans*, it was shown that worm NE-associated chromosomal domains tended to occupy the distal 3–5 Mb of the autosomal chromosomes, where gene density is low and repetitive elements are enriched [71]. Interestingly, most LADs — particularly in fly and mammalian cells — have sharp borders, with specific sequence elements that contain binding sites for the insulator protein CTCF and YY1 [69, 72] (reviewed in [11]).

Several studies have monitored the progressive association of repressed pluripotency genes and silent tissue-specific genes with the nuclear lamina during differentiation [68, 70, 71]. In the mouse ESC differentiation system, the percentage of the genome that was attached to lamin was high (40–48 %) [69], and only approximately 1000 (12 %) of over 17,000 genes scored showed a significant increase in lamin association during the commitment to neurons [70]. Importantly, these 1000 are enriched for pluripotency genes, which become repressed as cells differentiate, and silent non-neuron tissue-specific genes. Nonetheless, 30 % of the genes that became lamin-bound did not change in expression, indicating that the nuclear periphery does not necessarily impose transcriptional repression [73, 74].

In the other direction, the correlation was more robust: many of the genes that were released from the lamina upon differentiation were shown to be ‘unlocked’ or ‘open’ for lineage-specific transcription, even though active transcription occurred only much later. This is consistent with a recent study that showed that it is sufficient to unfold chromatin to provoke the shift of a promoter away from the nuclear lamina in mouse ESCs [75]. The authors induced chromatin de-condensation by targeting an acidic peptide, and found that this triggered release from the nuclear periphery for three developmentally regulated loci, in the absence of transcriptional activation [75]. This is reminiscent of results observed by DamID [70], which showed that genes expressed in terminally differentiated neurons shifted away from the nuclear periphery without increasing transcription in the committed precursor state (NPC), although the genes do become activated later [70]. Similarly, in early worm development, the inward shift away from the nuclear periphery of a heterochromatic transgene containing *pha-4*, a marker of endoderm differentiation, occurred before its activation [76]. Finally, even though a comparison of LMN-1 DamID profiles from *C. elegans* embryos and adults showed significant concordance, tissue differentiation in adults was associated with an increased separation between NE-bound and NE-excluded regions [77]. Collectively, these results argue that release from the lamina might correlate with chromatin remodeling, rather than active transcription. This nonetheless supports the hypothesis that gene positioning and tissue specification are coupled.

## The importance of histone modifications in positioning heterochromatin

### H3K9 methylation

**Fig. 2:**
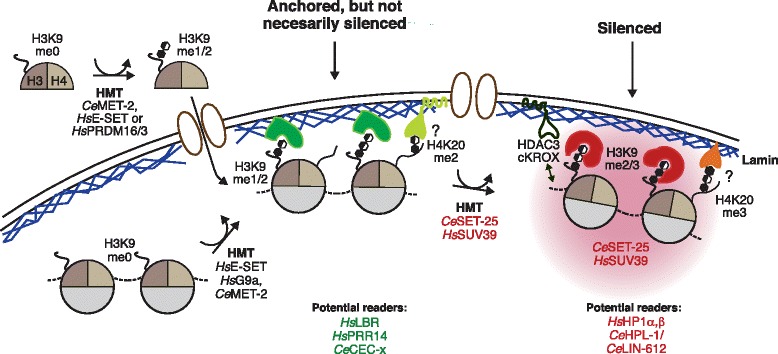
Histone modifications regulate perinuclear sequestration. A model of known and suggested histone tail modifications involved in heterochromatin anchoring at the nuclear envelope. The deposition of histones carrying H3K9^me1^ or H3K9^me2^ could be sufficient to ensure localization at the nuclear envelope according to work with the worm *Caenorhabditis elegans* [74]. Potential methyl readers that might contribute to anchoring include the lamin B receptor (LBR) in mammals and a *C. elegans* chromodomain protein (CEC-x) in worms. Readers of the H3K9^me3^ modification that ensure silencing include worm homologs of heterochromatin protein 1 (HP1) and LIN-61. Other factors implicated in tissue-specific gene repression and sequestration include cKROX and HDAC3, or an unknown reader of H4K20me3. See text for further details

To go beyond a simple correlation of H3K9 methylation and heterochromatin anchoring, genetic approaches are needed. The most extensive screen for factors involved in sequestering chromatin at the NE was a genome-wide RNA interference (RNAi) screen in *C. elegans* [74]. Using an integrated heterochromatic reporter, the Gasser laboratory identified two HMTs — MET-2 and SET-25 — as essential factors for the anchoring of heterochromatin to the NE in embryos. The first enzyme, MET-2, is the homolog of the mammalian histone-lysine N-methyltransferase SETDB1 (ESET), whereas SET-25 has a SET domain very similar to that of histone-lysine N-methyltransferase G9a (EHMT2), but lacks homology outside this region [74]. MET-2 and SET-25 work in a stepwise fashion, exclusively modifying histone H3K9 by depositing mono- (MET-2), di- (MET-2) and tri-methylation (SET-25). The *met-2 set-25* double-mutants lack all H3K9 methylation in embryos and during somatic cell differentiation, which not only de-represses a heterochromatic reporter but releases both it and endogenous H3K9^me^-enriched chromatin from the nuclear periphery, as mapped by lamin-DamID [71, 74] (Fig. 2). This links H3K9 methylation causally to chromatin anchoring, at least in early worm development.

In worms, the single *set-25* or single *met-2* mutants also shed light on the relationship between anchoring and transcriptional repression — neither mutation compromised perinuclear positioning of the heterochromatic reporter, but both individually led to its de-repression. As the *set-25* mutant strain lacks H3K9^me3^ but maintains wild-type levels of H3K9^me1^ and H3K9^me2^, H3K9^me2^ can clearly mediate anchoring, whereas H3K9^me3^ is needed for repression (Fig. 2). Thus, anchoring is not sufficient for silencing, yet the two are sequential events, both dependent on H3K9 methylation [74]. Other modifications or protein binding sites might act as a pre-requisite for H3K9^me^-mediated anchoring, although the *set-25 met-2* double-mutant did not alter methylation levels on histone H3 or H4 other than on H3K9 [74]. Intriguingly, worms that lack all H3K9 methylation are viable and differentiate to adulthood, although the *set-25 met-2* double mutants are sterile owing to impaired oogenesis at elevated temperature (e.g., at 25 °C; P Zeller, J Padeken and SMG, unpublished data).

When mammalian LADs were mapped in vivo, 80 % were enriched for H3K9^me2/me3^ [29, 70]. Moreover, reduction of the relevant H3K9^me2^ HMT, G9a, reduced but did not ablate lamin interaction, particularly of constitutively bound LADs [78]*.* While this suggests a positive role for H3K9^me2^ in perinuclear anchoring of chromatin in mammalian cells, another study based on fluorescence in situ hybridization (FISH) showed that mutation of G9a did not affect the localization of most tested lamin-bound loci in mouse ESCs, even though H3K9^me2^ levels were strongly reduced [79]. Harr and colleagues showed a significant drop in lamin association of an integrated, heterochromatic transgene in mouse cells upon G9a inhibition, although not a complete release [72]. The source of these discrepancies may lie in the method used to score the ‘anchoring status’ (FISH and microscopy versus DamID), or the fact that different cell types were used in each system. Taken together with the worm results, however, it appears that H3K9^me1^ or H3K9^me2^ has a conserved role in perinuclear heterochromatin anchoring, even if it is unlikely to be a sufficient signal in mammalian cells. Histone H3K9 methylation is not the only perinuclear targeting signal in worms either, since a second, H3K9^me^-independent, anchoring system has been shown to be induced in differentiated larval and adult tissues, to anchor heterochromatin (DSC and SMG, unpublished data). Thus, in both worms and mammals, anchoring pathways depend upon the differentiation state of a cell.

Part of the difficulty in defining the role of H3K9^me^ in heterochromatin anchoring in mammals is that this modification accumulates on centromeric satellite repeats and serves an essential role in kinetochore function [80]. Because of this, H3K9^me^ depletion leads to chromosomal missegregation in mitotically dividing mammalian cells. In contrast, holocentric worms (where the entire length of the chromosome acts as the centromere) have no mitotic defects in the absence of H3K9^me^ (J Padeken, personal communication). Moreover, HMT functions appear to be more redundant in the mammalian systems: not only can HMTs G9a and both Suv39H1 and Suv39H2 deposit H3K9^me2/me3^, but, in cells lacking both isozymes of Suv39H, centromeres lose H3K9^me3^ yet remain clustered owing to a compensatory function of H3K9^me1^, which accumulates at the centromere [80, 81]. In this case, the persistent satellite DNA architecture was thought to stem from the action of two H3K9-specific mono-methyltransferases, PRDM3 and PRDM16 [81]. Their simultaneous downregulation caused dispersal of centromeric foci and an accumulation of major satellite transcripts [81]. For other mammalian cell phenomena, such as the peripheral nuclear positioning of the β-globin locus on a bacterial artificial chromosome, localization was dependent on both Suv39H-mediated H3K9^me3^ and G9a-mediated H3K9^me2^ [82]. Consistently, in the study of an inducible LAD, Harr and colleagues found that knockdown of Suv39H1 or the prolonged treatment with an inhibitor of G9a reduced, but did not completely eliminate, perinuclear association in mouse fibroblasts, coincident with a reduction in both H3K9^me2^ and H3K9^me3^ [72]. Intriguingly, in this induced anchoring situation, polycomb-deposited H3K27^me3^ was also implicated in reporter association with the lamina [72].

### Anchors for heterochromatin

Assuming that histone H3K9 methylation, either alone or together with other modifications, targets sequences to the NE, it remains unresolved what factor(s) recognize the anchoring signal. It is unlikely that lamins bind specific lysine-methylated residues directly — rather, this is a job for specific ‘reader’ proteins that contain structurally defined chromo, PHD, MBT or tudor domains [83]. In HeLa cells, a previously uncharacterized proline-rich protein named PRR14 localizes to the nuclear envelope and promotes attachment of H3K9^me3^-marked heterochromatin, presumably through its interaction with the H3K9^me^ reader HP1 [84]. Interestingly, knockdown of PRR14 impairs myoblast differentiation [85], yet no specific loci were shown to be displaced from the NE in these cells. Future research should test the PRR14 anchoring function by means of quantitative binding assays for specific loci. However, knockout alleles encoding HP1α or HP1β in mice eliminate neither heterochromatin formation nor its localization [43]; moreover, in *C. elegans* embryos, the double deletion of genes encoding HP1 homologs (*hpl-1* and *hpl-2*), even in combination with loss of a third H3K9^me^ reader, LIN-61, left heterochromatic reporters anchored [74]. It is thus highly likely that additional H3K9^me^-recognizing anchors exist.

In mammals, one of these anchors could be the lamin B receptor LBR, which co-immunoprecipitates with H3K9^me3^-marked chromatin [86] and appears to interact with HP1 [87]. Unlike the worm LBR homolog, mammalian LBR has a C-terminal tudor domain that binds to H4K20^me2^ in vitro [88]. Unfortunately, H4K20^me2^ is distributed broadly across the genome, without significant enrichment in LADs [89], and H4K20^me3^ is enriched in centromeric satellite DNA, which does not always bind the nuclear envelope [90]. Moreover, in s*uv420h2* knockout mice, which have strongly compromised levels of H4K20^me3^, peripheral heterochromatin appears normal [91]. Nonetheless, given the genetic evidence that LBR is crucial for heterochromatin anchoring in some differentiated mouse tissues [8], it will be important to test for a combinatorial effect(s) or redundancies between H4K20^me2^ and HP1 in mammalian heterochromatin anchoring.

In *C. elegans*, targeted RNAi and mutagenesis screens aimed at identifying factors that compromise heterochromatin anchoring in either embryos or differentiated tissues have been performed. In embryos, a novel H3K9^me^ reader appears to mediate anchoring in embryos (A. Gonzales-Sandoval and SMG, personal communication), whereas in differentiated tissues methylation marks other than H3K9 contribute to heterochromatin anchoring. These differentiation-induced alternative pathways, along with a lack of centromeric heterochromatin, are the likely explanation for the near-normal development of H3K9-deficient worms [74].

### Alternative heterochromatin anchoring pathways and redundancy

Preliminary data address the nature of these alternative, differentiation-driven and H3K9^me^-independent, pathways for chromatin anchoring. The polycomb-deposited mark H3K27^me3^ is a plausible candidate as it marks facultative heterochromatin, particularly at developmentally regulated promoters [92], and is enriched in the outermost borders of LADs [69]. Recent work in mouse 3 T3 MEFs showed that H3K27^me3^ contributes to the peripheral relocation of a sequence located at the edge of a LAD [72], whereas, in worms, ablation of PRC2 components *mes-3* and *mes-6* leads to de-repression of a heterochromatic reporter in embryos and differentiated tissues, but no release from the NE [74]. Moreover, most polycomb-positive or H3K27^me3^-positive foci in differentiating cells are not perinuclear [91]. In uncommitted cells, this might stem from the coupling of H3K27^me3^ with H3K4^me3^, a mark that is actively excluded from the NE.

Further evidence for redundant, accumulative, but also alternative, pathways of heterochromatin tethering came from an elegant study that examined nuclei in differentiated tissues of wild-type mice and mice deficient for LBR and/or lamin A/C [8]. In the most extreme cell type studied, that of wild-type rod photoreceptor cells of the retina, the authors found an ‘inverted’ spatial organization of chromatin, with heterochromatin in the nuclear core and euchromatin at the periphery [7]. They showed that this inverted topology was due to the fact that neither LBR nor lamin A/C are expressed in these highly specialized retinal cells [8]. LBR is typically induced earlier in tissue development than lamin A/C, and, in tissues where both are expressed, or when one is ectopically expressed to replace the other, a ‘conventional’ nuclear architecture was restored. Intriguingly, the artificial induction of LBR in these retinal cells, but not lamin C, was sufficient to keep heterochromatin at the nuclear periphery, arguing that lamin A/C requires other proteins or chromatin ligands for interaction [93]. The missing factor(s) was not any of the known INM lamin-interacting proteins [8]. By contrast, knowing that LBR-deficient embryonic stem cells are viable, it should be possible to devise a screen for restored heterochromatin anchoring in mice.

Finally, sequence-specific binding sites might also play a role in locus-specific lamin attachment, as evidenced in a study of the *IgH* LAD in mice [94]. In this study, a GAGA motif binder, cKrox, was shown to bind to HDAC3 and Lap2β, a lamin-associated gene regulator. Lap2β shows selective anchoring activity that is cell type- and developmental stage-specific. HDAC3 appears to be a recurrent factor in NE tethering as it also binds to emerin [95, 96]. However, neither Lap2β nor HDAC3 can account for the widespread association of heterochromatin with the NE. It is likely that different loci use different anchoring pathways in differentiated cells, as observed in worms. Indeed, a comparison of lamin and emerin DamID profiles in *C. elegans* showed that, despite an overlap of 89 % between DamID profiles, these two NE proteins also were bound by different sets of tissue-specific genes [77]. The dominance of one anchoring mechanism over another for a given locus might depend on aspects of the local chromatin state, the presence of *cis*-acting elements, proximity to developmentally regulated promoters, and possibly on cell-type variations in the composition of the NE [59, 97].

Independent of these heterochromatin pathways, there is a conserved DNA tethering mechanism that relies on the SUN domain family of anchors (named derived from the *Schizosaccharomyces pombe* Sad1 and *C. elegans* Unc-84 proteins), a class of NETs that are anchored both by lamins (in vertebrates) or by interaction with chromatin (in yeast). In the intermembrane space, their C-terminal SUN domain interacts with nesprins, which extend through the outer nuclear membrane to the cytoskeleton (reviewed in [98, 99]). This so-called LINC complex (‘linking inner nuclear membrane and cytoskeleton’) has been implicated in chromatin tethering from yeast to human, but, most notably, it functions universally in the formation of the meiotic ‘bouquet’ structure in which telomeres are clustered to promote homolog pairing before the pachytene stage. SUN domain proteins in yeast and worms also help anchor telomeres in mitotic cells [100–102]. Intriguingly, mutation of nesprins, which link to the cytoskeleton, or perturbation of the level of SUN domain proteins, leads to defects in the function of human differentiated tissues, such as those of the inner ear [103].

## Self-reinforcing mechanisms that sequester silent chromatin at the nuclear periphery

During cell differentiation, uncommitted cells with identical genetic information acquire epigenetic changes that need to be passed on through mitotic cell division in order to maintain lineage specification. Current models for the epigenetic inheritance of histone methylation propose that HMTs are recruited to chromatin by the marks they deposit, thus ensuring both the modification of neighboring nucleosomes and the propagation of the mark onto newly deposited nucleosomes at the replication fork. Good support for this mechanism exists for the propagation of H3K27^me3^ by PRC2 [104], for the spreading of H3K9^me3^ in fission yeast by Clr4 [105], for the maintenance of H3K9^me3^ at centromeric repeats in mammals by Suv39 [106, 107] and of H3K9^me2^ by G9a [108, 109]. Similarly, in *C. elegans*, SET-25 becomes enriched in foci that colocalize with the mark it deposits, H3K9^me3^, in a manner that is independent of the HP1 homologs, even though the worm Hpl-1 and Set-25 proteins colocalize in heterochromatic foci.

The fact that H3K9 mono- and di-methylation is a trigger for perinuclear chromatin anchoring suggests that the pathway towards heterochromatin can itself drive its spatial segregation from active chromatin domains. Furthermore, the finding that the HMT that deposits the terminal, repressive H3K9 methylation mark remains bound to perinuclear heterochromatin explains how the nuclear periphery is favorable for both the establishment and propagation of repression. This circularity could act as a self-reinforcing mechanism that ensures the robust separation of active and inactive chromatin domains.

We note that a similar mechanism has been demonstrated for SIR-mediated silencing in budding yeast, where peripheral anchoring is mediated by the chromatin-bound Sir4 protein (reviewed in [21]). Sir4 is required to nucleate repression, through the recruitment of both Sir2 (to deacetylate H4K16ac) and Sir3 (which binds to deacetylated histones to repress transcription), and then Sir4 remains bound as an integral component of silent chromatin. Sir4 also ensures the tethering of silent chromatin to the yeast NE, and targeted Sir4 is sufficient to shift an active locus to the yeast nuclear periphery [110]. This is conceptually analogous to the situation in *C. elegans*, where H3K9^me1/me2^-containing chromatin binds to the NE before establishment of the repressed state. Thus, peripheral sequestration of chromatin both nucleates and propagates repression. Given that peripheral attachment also favors late replication [36, 51], the timing of replication of peripheral chromatin might further reinforce heritable repression.

## The functional implications of gene positioning

It is clear that the NE cannot be considered exclusively as a repressive compartment, nor is the nuclear interior uniformly active. Nonetheless, elegant gain-of-function targeting assays show that subnuclear compartments can influence gene expression. In particular, the tethering of genes to repressive zones of the NE, notably to NE-bound telomere clusters in yeast or to emerin/lamin zones in mammals, can facilitate gene repression (reviewed in [111]). In mouse fibroblasts, some tethered genes responded to positional cues and others did not — this variability reflecting the strength of the promoter and the integration site of the reporters in the genome. Indeed, a high-throughput analysis of 27,000 reporter integrations in the genome of mouse ESCs showed that expression levels vary significantly depending on the integration site, but also confirmed that most reporters integrated into LADs have lower transcription levels [112]. The conclusion from these studies is that, although the NE can favor repression, position alone is not sufficient to repress a gene, nor does transcription per se drive a gene away from the periphery (Fig. 2).

Nonetheless, by now a large number of examples show the relocation of a transcriptionally active, developmentally regulated gene from the NE to the interior lumen of the nucleus in a tissue-specific or cell type-specific manner (reviewed in [113]). In several organisms, including *C. elegans* [114], developmentally regulated promoters have been observed to move upon activation from a random or peripheral distribution to the nuclear interior, even overcoming a methylated H3K9 heterochromatic state [115].

An exception to this trend of shifting inwards during activation is the major heat-shock gene, a conserved gene homologous to *HSP70* in human. In *C. elegans*, this locus (*hsp16*.2) is found juxtaposed to nuclear pores, independent of its expression status, and the gene becomes even more tightly associated with nuclear pores upon induction of heat shock [116]. In flies, as in yeasts and worms, the association of stress-induced genes with nuclear pores requires components of the regulatory SAGA complex and the RNA processing and export machinery THO-TREX [117]. This might also be the case for the upregulated male X chromosome in *Drosophila* [118, 119]. Whether this mechanism controls RNA turnover and export, or promoter efficiency, remains unclear.

## Chromatin organization and lamins

In general, the integrity of the inner nuclear envelope is important for stable gene expression. This was shown for a heterochromatic array in *C. elegans* following depletion of the lamin homolog LMN-1, in *Drosophila* testis, and finally in mammalian cells lacking lamins or associated components [115, 120–122]. Lamin depletion, however, impacts many other nuclear processes, making it impossible to conclude that lamin association directly controls gene expression. More compelling evidence for the role of lamins in the spatial organization of the genome and its expression comes from the study of specific point mutations in lamin A or in its associated proteins emerin, Lap2β and Man1, which cause various late-onset degenerative diseases in humans, collectively called laminopathies [10] (Table 2).

**Table 2:** Classification of the laminopathies^a^

Affected tissue/phenotype	Disease	Full name/description	OMIM code
Muscle	EDMD2	Autosomal-dominant Emery–Dreifuss muscular dystrophy	#181350
	EDMD3	Autosomal-recessive Emery–Dreifuss muscular dystrophy	#604929
	LGMD1B	Limb girdle muscular dystrophy type 1B	#159001
	CMD1A	Dilated cardiomyopathy 1A	#115200
	CCD	Cardiac and conduction defect	
	AD-SMA	Autosomal-dominant spinal muscular atrophy	
	LAF	Lone atrial fibrillation	
		Generalized muscular dystrophy and/or cardiomyopathy phenotype	
		dropped head syndrome	
Fat	FPLD1	Familiar partial lipodystrophy TYPE 1	#608600
	FPLD2	Familiar partial lipodystrophy TYPE 2	#151600
		Generalized lipodystrophy phenotype	
Neuronal	CMT2B1	Charcot-Marie-Tooth type 2B1	#605588
		Generalized neuropathy phenotype	
Multisystem	MADA	Mandibuloacral dysplasia	#248370
	RD	Restrictive dermopathy	#176670
		Generalized metabolic syndrome phenotype	
		Slovenian type heart-hand syndrome	
Premature aging	HGPS	Hutchinson–Gilford progeria syndrome	#176670
	WRN-like	Atypical Werner syndrome	#277700
	LIRLLC/LDHCP	Generalized lipoatrophy, insulin-resistant diabetes, disseminated leuko-melanodermic papules	#608056
		Liver steatosis and cardiomyopathy	

^**a**^List of human genetic diseases and disorders caused by mutations in the *LMNA* gene, classified by type of tissue affected (see also [151])

Most laminopathies are autosomal dominant and generally cause late-onset degeneration of either striated muscle, heart, adipocytes, peripheral neurons, skin or bones, with only a few mutations leading to systemic progeria [10, 55, 123]. Currently, over 460 different disease mutations have been mapped to the human *LMNA* gene, defining 17 distinct diseases, more than in any other human gene [124] (Table 2; Fig. 1). Various models have been proposed to explain how a single *LMNA* gene can generate so many distinct pathologies. It has been proposed that lamin mutations affect gene expression in a tissue-specific manner, possibly by influencing perinuclear chromatin organization. In some cases, there appear to be defects in repair of DNA damage or loss of function of adult stem cells, whereas, in yet others, the nucleus becomes unable to resist mechanical stress (a common feature of diseased muscle tissue) or mechanotransduction signaling is compromised, thereby perturbing cell differentiation (for reviews, see [10, 55]). Clearly, these models are not mutually exclusive.

One frequent pathology arising from lamin A/C mutations is the autosomal-dominant Emery–Dreifuss muscular dystrophy (AD-EDMD) [10], which can also arise through mutation of the lamin-binding protein emerin (X-linked EDMD). This is consistent given that lamin A is necessary for proper localization of emerin at the nuclear periphery [66, 125]. However, not all AD-EDMD mutations cause displacement of emerin [126, 127], and it is difficult to explain why a loss of emerin binding would be autosomal dominant. Intriguingly, most mouse models of the human laminopathic mutations fail to recapitulate their autosomal-dominant features, restricting the use of mouse as a model system.

A genetic study of a specific AD-EDMD mutation in *C. elegans*, by contrast, has suggested that hyper-sequestration of genes at the nuclear lamina leads to a dominant, striated-muscle defect [128]. The ectopic expression of the Y45C point mutation introduced into *C. elegans LMN-1* (Y59C) led to an inability to release muscle-specific genes from the NE in muscle tissue at a stage when these promoters should normally be induced. Although the muscles could still develop, the tissue was misorganized, and there was a noticeable loss of muscle function in adult worms [128]. If muscle-specific genes are inappropriately expressed owing to peripheral sequestration, causing the disease phenotypes, then interference in heterochromatin sequestration might be a plausible treatment for AD-EDMD patients. This model would explain the gain-of-function, dominant-negative character of this particular mutation.

A further laminopathic *LMNA* allele that has been studied in detail in both mouse and *C. elegans* encodes a protein lacking lysine 32 (ΔK32). Mice homozygous for the ΔK32 *LMNA* mutation show a delay in maturation of striated muscle and have metabolic defects that include reduced adipose tissue and hypoglycemia, which leads in turn to premature death. The transcription factor SREBP-1, which was previously shown to interact directly with lamin A protein [129], showed reduced activity in the ΔK32 knock-in mice, causing liver failure and death [130]. In *C. elegans*, the equivalent mutation (ΔK46) caused alterations in the in vitro lateral assembly of dimeric head-to-tail lamin polymers, which is a prerequisite step for the formation of filaments. This led to an abnormal organization of lamin protofilaments and a decreased affinity for emerin in vitro [131]. Remarkably, in *C. elegans*, the ΔK46 mutation caused lamin aggregation with LEM-2 in vivo, and emerin displacement to the cytoplasm, and provoked motility defects and abnormalities of muscle structure [131].

*Drosophila* has provided another genetic model for lamin deficiencies. *Drosophila* larval cells lacking the A-type lamin C have NE defects, including changes in nuclear morphology and the clustering of nuclear pore complexes, much like those observed in human laminopathies [132]. Ectopic expression of a mutant lamin C lacking its first 42 amino acids (head domain) caused muscle defects, abnormal organization of the cytoskeleton and disrupted muscle striation [133]. The small fraction of animals that managed to escape larval lethality had leg defects, consistent with a loss of muscle function and ecdysone hormone signaling [133]. In both worms and flies, other missense AD-EDMD-linked mutations caused lamin aggregation, although most had no visible adult phenotypes.

A wide range of mutations in lamin A/C have been correlated with changes in higher-order chromatin organization, and particularly severe effects accompany the C-terminal deletion that provokes systemic progeria, or Hutchinson Gilford progeria syndrome. It is unclear whether its chromatin effects cause or result from the premature-aging phenotypes as the progeria mutation also affects cell metabolism and WNT and NOTCH signaling [134, 135]. Interestingly, embryonic fibroblasts derived from mouse models of this disease do not show early senescence, whereas adult fibroblasts do; senescence was traced to the inability of the adult fibroblasts to produce a functional extracellular matrix, which in turn reduced WNT signaling, promoting early senescence [136].

Less-dramatic phenotypes arise from lamin point mutations that appear to cause a loss or gain of interaction with specific transcription factors. One well-studied case is that of SREBP1, a transcription factor that binds to the sterol regulatory element on DNA and regulates the genes required for de novo lipogenesis. SREBP1 is a lamin A binding partner in mouse adipocytes, and lipodystrophy-linked mutations map to the SREBP1-binding domain in lamin A/C. Inappropriate sequestration or improper release of SREBP1 might thus be responsible for the fat loss seen in patients carrying these mutations. In a further exciting study, lamin A/C and emerin were shown to regulate the nuclear localization of the mechanosensitive transcription factor myocardin-like protein 1 (MKL-1, also known as MAL or MRTF-A), possibly by modulating the balance between G-actin and F-actin [137]. Indeed, emerin caps pointed-end actin filaments and could modulate actin dynamics at the NE [138]. If defective, this might lead to an inability to cope with mechanical stress.

Other transcription factors that associate with lamin or lamina-associated proteins include germ-cell-less (GCL), which binds to the INM protein LAP2β in mouse, and the DP3 subunit of the E2F-DP3 heterodimer, which influences the regulation of E2F-dependent genes [139]. The transcription factor Oct-1 is localized to the nuclear lamina and represses the aging-associated collagenase gene at the NE. In aging cells, it loses this association, and the collagenase gene becomes active [140]. In addition, the inner-membrane spanning protein MAN-1 binds to SMAD4, which in turn brings regulatory SMADs to the nuclear periphery to inhibit the bone morphogenetic protein 4 (BMP4) signaling pathway [141]. Finally, several LEM-domain proteins (e.g., LAP2β and emerin) bind to the small transcription regulator barrier to autointegration factor (BAF), as well as the histone deacetylase HDAC3 and HA95 (reviewed in [142]). Exactly what roles these factors play in gene expression is still unclear, but HDAC3 and BAF have both been associated with mammalian promoters. Given that there are known instances in which the mislocalization or sequestration of a transcription factor perturbs gene activation (e.g., [143]), it is not difficult to imagine a mechanism through which a mutant lamin A fails to bind, or fails to release, a given transcription factor, leading to gene misregulation. Substantiating such mechanisms in differentiating human tissues, however, will be a difficult task.

## Dealing with redundancy as one goes forward

Clearly, there is much left to discover about how nuclear lamins and nuclear positioning affect tissue-specific gene expression, yet in all cases it is necessary to demonstrate causality and not simply correlation. Future research must focus on the crucial link between chromatin states and NE partners, while dealing with the redundancies that we know exist among factors that anchor chromatin in the interphase nucleus. Clever screens in organisms that are partially compromised for aspects of nuclear organization should provide the means to identify essential components of other redundant pathways. Forward-genetic screens for dominant, gain-of-function phenotypes will also be needed to verify new components. Deciphering the mechanisms that determine the spatial organization of the genome in differentiated tissues requires that one monitors tissue-specific spatial distributions, which presents a challenge for high-throughput genetic approaches, yet clues can be gained from human diseases that affect nuclear organization. Fortunately, chromatin modifications and NE proteins — with the exception of the absence of lamin in plants and yeast — appear to be some of the most highly conserved proteins in our genomes. Thus, it is likely that we will be able to discover and test new molecules involved in the organization of the interphase genome through development and tissue-specific differentiation by capitalizing on trans-species studies of nuclear organization.

## Box 1. Approaches used for the analysis of nuclear organization

Imaging approaches

The use of microscopy has the advantage of revealing the spatiotemporal localization of a defined genetic locus in the nucleus in relation to other landmarks at a single-cell level.

- Fluorescence in situ hybridization (FISH)
  FISH is based on the hybridization of fluorescent probes with specific DNA, RNA or whole-chromosome sequences. Drawbacks include artifacts that might arise during the fixation steps of cells and/or tissues and the denaturation of DNA that is required for hybridization.
- LacO/LacI–GFP or TetO/TetR–GFP
  In order to analyze the position and dynamics of chromatin loci in living cells, arrays of bacterial operators can be integrated at a site of interest and the corresponding bacterial ligand, fluorescently labeled with green fluorescent protein (GFP), is expressed constitutively at low levels (e.g., the lactose (lac) operator *LacO* together with the labeled lac repressor LacI–GFP, or the tetracycline (tet) operator *TetO* together with the labeled Tet repressor *TetR–*GFP [110]). Drawbacks can be secondary effects of repressor binding repeats, although this can be avoided by using a mutated form of LacI that binds less tightly [144].
- Other fluorescence-based applications
  The fusion of fluorescent proteins to specific nuclear proteins can also be used to monitor chromatin dynamics and nuclear organization. However, one must always test for genetic complementation by the fusion protein. Photoactivation of labeled histones at specific nuclear compartments allows the determination of subnuclear localization of the perinuclear chromatin after cell division (e.g., see [145]). Imaging of histone modifications in living cells is also becoming achievable thanks to new methods such as FRET-based sensors or injection of fluorescently labeled histone-specific modified antibody (Fab) fragments (reviewed in [146]). The extension of these methods to super-resolution microscopy will provide an even more detailed understanding of the nuclear organization.

3C/4C/5C/HiC methods

The chromosome conformation capture (3C) technique and various derivative methodologies (4C, 5C, HiC) enable low-resolution analysis of DNA–DNA interaction probabilities, over approximately 10 kb to roughly 1 Mb. This technology uses crosslinking, enzymatic digestion, ligation, amplification and determination of the interactive sequences by PCR or deep sequencing [15]. 3/4/5C analysis can reveal the contacts between a gene of interest and its regulatory elements found intra-chromosomally or inter-chromosomally, whereas the HiC method can reveal ‘all versus all’ genomic interactions (e.g., [147]).

DNA adenine methyltransferase-fusion identification (DamID)

The DamID technique is an alternative method for detecting protein–DNA contacts based on fusing a chromatin or nuclear protein of interest to *Escherichia coli* DNA adenine methyltransferase (dam), which leads to preferential methylation of GATC motifs that are in the vicinity of the fusion protein. The sequences become differentially sensitive to restriction enzymes, allowing their selective amplification for detection by microarrays or deep-sequencing [16, 148]. Variations on this theme include inducible and time-resolved DamID methods.

Chromatin immunoprecipitation (ChIP) variants: ChIP-chip/ChIP-seq/ChIA-PET

These approaches are used to investigate interactions between proteins or specifically modified proteins and DNA in vivo and at a genome-wide level. The ChIP-chip, ChIP-seq and ChIA-PET methods are based on the recovery of DNA that is crosslinked to a specific antigen of interest, followed by microarray, high-throughput sequencing or 3C technology [15]. The ChIP-chip and ChIP-Seq techniques are also commonly used to study the genome-wide distributions of epigenetic marks. Additional approaches to study epigenomics such as MeDIP-seq, Methyl-Cap-seq, RRBS and Infinium have been developed to map DNA methylation at the genome level (for review, see [149]). Genome-wide bisulfate sequencing has allowed base-pair resolution and quantitative estimates of CpG methylation by methyl-cytosine (meC) chemical modification [14].

Genetic approaches: gain of function, loss of function and spatially targeted function

To test for correlations between position and function revealed by the above-mentioned methods, one needs to perturb normal function. Classically, truncations, frameshifts or deletions of genes provide loss-of-function data, whereas gain-of-function mutations or fusion proteins help confirm that the effects are not indirect. One commonly used gain-of-function example is the targeting of a specific protein or DNA locus to a nuclear subcompartment, accompanied by monitoring the resulting changes in function [110, 150].

## Abbreviations

AD-EDMD: autosomal-dominant Emery–Dreifuss muscular dystrophy
BAF: barrier to autointegration factor
ChIP: chromatin immunoprecipitation
DamID: DNA adenine methyltransferase (dam)-fusion identification
ESC: embryonic stem cell
FRAP: fluorescence recovery after photobleaching
FRET: fluorescence resonance energy transfer
HDAC: histone deacetylase
HGPS: Hutchison Gilford progeria syndrome
HMT: histone methyltransferase
HP1: heterochromatin protein 1
INM: inner nuclear membrane
LAD: lamin-associated domain
LBR: lamin B receptor
LEM: lamin, emerin, Man1
LINC: linking inner nuclear membrane and cytoskeleton
MBT: malignant brain tumor
meC: methyl-cytosine
NE: nuclear envelope
NET: nuclear envelope transmembrane protein
NPC: neural precursor cell
Pc: polycomb
SAGA: Spt3, Ada2, Gcn5, Ada3 complex
SIR: silent information regulator
SUN: *S. pombe* Sad1, and *C. elegans* Unc-84 related
